# The thalamus and basal ganglia are smaller in children with epilepsy after perinatal stroke

**DOI:** 10.3389/fneur.2023.1252472

**Published:** 2023-09-28

**Authors:** Ulvi Vaher, Norman Ilves, Nigul Ilves, Rael Laugesaar, Mairi Männamaa, Dagmar Loorits, Pille Kool, Pilvi Ilves

**Affiliations:** ^1^Department of Radiology, Institute of Clinical Medicine, University of Tartu, Tartu, Estonia; ^2^Children's Clinic, Tartu University Hospital, Tartu, Estonia; ^3^Radiology Clinic, Tartu University Hospital, Tartu, Estonia; ^4^Department of Pediatrics, Institute of Clinical Medicine, University of Tartu, Tartu, Estonia

**Keywords:** ischemic perinatal stroke, epilepsy, interictal epileptiform discharges, thalamus, basal ganglia

## Abstract

**Background:**

Epilepsy is one of the most serious consequences of perinatal stroke. Epilepsy itself has been proposed as a risk factor for impaired cognitive, language, and behavioral functioning. It is still unclear which children develop epilepsy after perinatal stroke. The current study aimed to evaluate the volume of the thalamus and the basal ganglia in children after perinatal stroke in relation to poststroke epilepsy.

**Methods:**

The follow-up study included 29 children with perinatal arterial ischemic stroke (AIS), 33 children with presumed periventricular venous infarction (PVI), and 46 age- and sex-matched healthy controls. Magnetic resonance imaging was performed in children between the ages of 4 and 18 years, and volumetric analysis by segmentation was used to evaluate the size of the thalamus, caudate nucleus, putamen, globus pallidus, hippocampus, amygdala, and nucleus accumbens.

**Results:**

During a median follow-up time of 12.8 years [interquartile range (IQR): 10.8–17.3] in the AIS group and 12.5 years (IQR: 9.3–14.8) in the PVI group (*p* = 0.32), epilepsy developed in 10 children (34.5%) with AIS and in 4 (12.1%) children with PVI, *p* = 0.036 [odds ratio (OR) = 3.8, 95%, confidence interval (CI): 1.04–14]. Epilepsy and interictal epileptiform discharges (IEDs) without clinical seizures were more often expressed in children with AIS (*n* = 16, 55%) than in children with PVI (*n* = 7, 21.2%), *p* = 0.0057 (OR = 3.8 95% CI: 1.04–14). In the AIS group, the ipsilesional and contralesional thalamus, ipsilesional caudate nucleus, and nucleus accumbens were significantly smaller in children with epilepsy compared to children without epilepsy. In the PVI group, the ipsilesional thalamus, caudate nucleus, and nucleus accumbens were smaller in the pooled group of epilepsy plus IED alone compared to children without epilepsy.

**Conclusion:**

In children with AIS, epilepsy or IED occurred more often compared to children with PVI. Both patients with AIS and PVI with severe damage to the basal ganglia and the thalamus have a higher risk of developing poststroke epilepsy and should be monitored more closely throughout childhood to initiate timely antiseizure medication and rehabilitation.

## Introduction

Perinatal stroke is a focal vascular brain injury occurring between 20 weeks of fetal life and the 28th postnatal day, with a growing overall birth prevalence of 1:1,100 among term-born children (1, 2). The most frequent vascular types of ischemic perinatal stroke are arterial ischemic stroke (AIS) and periventricular venous infarction (PVI) (2–4).

More than 50% of children have adverse outcomes after ischemic perinatal stroke (5–7). Epilepsy is one of the most serious consequences, and it develops in 6–71% of patients with perinatal stroke. The wide range of incidence rates depends on the studied vascular subtype and the length of follow-up (8–11). A recent meta-analysis found a summary epilepsy incidence of 27.2% over a mean follow-up time of 10.4 years in perinatal AIS (12). Epilepsy occurs more often after AIS compared to PVI (3, 8). The risk of developing epilepsy after an ischemic perinatal stroke is highest in the first 6 months but is still elevated in young adults (11). However, it is not yet clear which children with perinatal stroke carry a higher epilepsy risk.

Epilepsy can be a key risk factor for impaired non-verbal intelligence after perinatal AIS and PVI (13). Sleep-related electroencephalographic (EEG) abnormalities, such as spike and wave discharges, occurring during >75% of sleep, have been associated with worse developmental outcomes in language and behavior in children with perinatal AIS (14). The parents of children with poststroke epilepsy have reported worse scores on physical, social, and school scales and on movement and fatigue dimensions compared with children without epilepsy (15). Therefore, it is important to predict which children with perinatal stroke may develop epilepsy.

Only a few studies have evaluated the size of damage to the deep brain structures in children with ischemic perinatal stroke. Volumetric investigations have revealed changes in thalamic volume after perinatal stroke in children with motor impairment (16, 17).

The predictive value of evaluation of the size of the thalamus and the other subcortical structures in the case of perinatal poststroke epilepsy has received comparatively little attention, and relevant data are scarce. Based on the visual assessment of magnet resonance images, poststroke epilepsy has been associated with more extensive cortical damage, multiple strokes, simultaneous cortical and basal ganglia impairment, and involvement of the thalamus and the temporal lobe (8, 9, 18). A study on infantile spasms after perinatal AIS identified the relationship between the development of spasms and infarct size and affected deep cerebral structures (caudate nucleus, nucleus lentiformis, capsula interna, and thalamus) (19). A reduction in hippocampal volume, both ipsilateral and contralateral to the lesion, has been described in one study of patients with epileptic seizures after AIS (20).

The current study aimed to evaluate the volume of the thalamus and the basal ganglia in children after ischemic perinatal stroke in relation to poststroke epilepsy. We hypothesized that the size of the thalamus, basal ganglia, and hippocampus is smaller in children with perinatal stroke who develop epilepsy compared to children who do not develop epilepsy and healthy controls.

## Materials and methods

This is an observational, regional, population-based, consecutive cohort study of patients with ischemic perinatal stroke. The study is part of a larger study on the outcome of children with perinatal stroke (4, 7, 8, 16, 21–24).

### Participants

Patients were identified from the Estonian Pediatric Stroke Database. Data were collected retrospectively within an epidemiological study from 1994 to 2003 and prospectively in 2004 (8, 21). All children with pediatric stroke admitted to the Children's Clinic of Tartu University Hospital, which is one of the two-third-level child neurology centers in Estonia, are included in the Pediatric Stroke Database and were invited to participate in the outcome study.

Based on the time of diagnosis, ischemic perinatal stroke is classified into fetal ischemic stroke, neonatal ischemic stroke, and presumed perinatal ischemic stroke (2). Neonatal ischemic stroke is diagnosed after birth, before or on the 28th postnatal day. Presumed perinatal ischemic stroke is diagnosed in infants >28 days of age after the uneventful neonatal period. Neuroimaging displays signs of chronic infarction in children with presumed perinatal ischemic stroke, and it is presumed that an ischemic event has occurred between the 20th week of fetal life and the 28th postnatal day (2, 4).

All radiological images [cerebral ultrasonography, computer tomography, and magnetic resonance imaging (MRI)] of the patients in the Estonian Pediatric Stroke Database, stored in the population-based Estonian Picture Archive, were reviewed independently by a radiology resident (No. I) and two neuroradiologists (PI and DL), who were all blinded to the clinical outcome of the patients. The diagnosis of perinatal stroke and the type of vascular genesis are based on a consensus agreement and previous classifications (3).

The study participants fulfilled all inclusion criteria: (1) MRI confirmed the diagnosis of unilateral perinatal stroke—neonatal AIS, presumed perinatal AIS or presumed perinatal PVI; (2) birth ≥36 weeks of gestational age; (3) follow-up 3T MRI scan including 3D T1-weighted imaging at the age of 6–18 years; and (4) clinical follow-up of at least 4 years. The exclusion criteria were as follows: (1) structural disease other than stroke affecting the central nervous system (hypoxic-ischemic encephalopathy, central nervous system infectious disease, tumor, cortical malformation, and congenital anomaly); (2) specific disease-causing gene variant or copy number variant suggested to be pathogenic for epilepsy or developmental delay; and (3) absence of an MRI confirming stroke.

The control group consisted of healthy volunteers of age- and sex-matched children recruited from the coworkers' relatives and children, as well as their classmates.

### Data abstraction

Clinical information about pregnancy, delivery, and the neonatal period was collected from medical records and patient interviews as reported earlier (4, 8, 23). Pregnancy and birth history, symptoms during the neonatal period, stroke presentation, age at the first epileptic seizure and age at the epilepsy diagnosis, seizure semiology, antiseizure medication, seizure control, and presence of status epilepticus were recorded.

Neonatal seizures were diagnosed by epileptiform activity on EEG and were defined as sudden, repetitive, evolving, and stereotyped episodes of abnormal electrographic activity with an amplitude of at least 2 μV and a minimum duration of 10 s (25), which might or might not be accompanied by clinical manifestations. In one patient from the AIS group, neonatal seizures were diagnosed based on clinical features alone.

Epilepsy is defined as a disease if it meets one of the following conditions: (1) at least two unprovoked seizures occurring >24 h apart; (2) one unprovoked seizure with high recurrence risk; and (3) a diagnosis of the epilepsy syndrome (26). In our study, epilepsy was often diagnosed after the first seizure if it occurred at least 1 month after stroke; EEG confirmed epileptiform activity concordant with seizure semiology and a pre-existing brain lesion, which all indicate a high risk for recurrence of seizures (26). All epilepsy diagnoses were reviewed and confirmed by a child neurologist and an experienced epilepsy specialist (UV).

EEG was performed for perinatal stroke patients as part of follow-up evaluation in case it had not been performed after a period of infancy. The standard EEG in the postneonatal period included the awake and sleep (daytime nap) periods. An international full 10–20 system was used for electrode placement (27).

Based on epilepsy diagnosis and EEG findings, the children with perinatal stroke were divided into three groups: (a) group of epilepsy—patients with a confirmed epilepsy diagnosis; (b) group of interictal epileptiform discharges (IEDs)—patients with epileptiform activity on EEG but without clinical seizures; and (c) group without epilepsy—patients without epilepsy or interictal EEG activity. A pooled group was formed on the basis of the epilepsy and IED groups.

Neurodevelopmental outcome was evaluated according to the Pediatric Stroke Outcome Measure (PSOM) (28). It contains five subscales: right sensorimotor, left sensorimotor, language production, language comprehension, and cognitive/behavioral performance. Each subscale yields a deficit severity score: 0—no deficit, 0.5—mild deficit, normal function, 1—moderate deficit, impaired function, and 2—severe deficit, missing function. The outcome was assessed by one of the two child neurologists (UV and RL), and the score at the last visit was used in the analysis.

### Neuroimaging

MRI for this study was performed in the chronic stage of perinatal stroke without anesthesia at the age of 6–18 years (*n* = 61), except for a single child with neonatal AIS at the age of 4.7 with anesthesia for MRI investigation for clinical purposes. A 3T Philips Achieva MRI scanner was used with an 8-channel SENSE head coil (Philips Medical Systems, Best, The Netherlands). Structural T1-weighted images with isotropic 1 × 1 × 1 mm voxels were obtained using a fast field echo sequence with TR = 8.2 ms, TE = 3.8 ms, and a field of view of 256 × 256 mm. Raw DICOM images were converted to the NIFTI format and anonymized by an investigator blinded to the clinical data (Ni. I).

### Volumetric analysis by segmentation

Analysis was performed using the FMRIB Software Library (FSL) (https://www.fmrib.ox.ac.uk/fsl/) version 6.0.5. The images of right-side lesions were flipped along the x-axis, and all the lesions were analyzed as left-side lesions. The automatic segmentation tool FSL FIRST (29) was used for the initial segmentation of the subcortical gray matter structures: the thalamus, caudate nucleus, putamen, globus pallidus, hippocampus, amygdala, and nucleus accumbens in both hemispheres.

Manual segmentation and quality control were needed to correct the faults induced by stroke-related morphologic changes in automatic segmentation. Manual segmentation was performed using the FSL's tool FSLeyes version 1.4.5 in a randomized subject order by a single investigator (Ni. I.) who was blinded to the study group and clinical outcome.

The volume of the segmented structures was measured using FSL's tool, fslstats. The measured volumes were normalized based on the individual's head size using an individual volumetric scaling factor developed by the FSL's SIENAX tool (30), which allowed the subject's brain volume to be converted to MNI152 standard space volume (16).

A subset of children was created for interrater and intrarater evaluation by selecting half (50%) of the children in each study group (AIS, PVI, and controls), using simple random sampling and taking account of the proportion of children with epilepsy. After 4 months, the manual segmentation of the subset was repeated by two investigators (Ni. I. and No. I.) who were blinded to the previous segmentations and to each other.

### Statistical analysis

First, the normality of the data was evaluated using the Shapiro–Wilk test. For each patient, inter- and intra-observer variation was calculated as the absolute value of the difference between two measurements divided by the value of the first measurement multiplied by 100 (31). Inter- and intra-observer reliabilities were estimated as the mean of the percentage of the relative difference with a volume range for the segmented structures. Continuous data were summarized as means with the 95% confidence interval (CI) or medians with the interquartile range (IQR), and categorical data, as absolute counts and percentages. The groups were compared using a one-way analysis of variance (ANOVA) *F-*test for continuous variables. Non-parametric alternatives (Kruskal–Wallis, Welch's ANOVA) were used when data were not normally distributed, or when variances were not equal between the groups. If significant, *post-hoc* pairwise comparisons were analyzed using the ANOVA Student's *t*-test method or the Mann–Whitney *U*-test for continuous variables (as appropriate). To compare proportions (qualitative variables), the chi-squared test and Fisher's exact test (when expected values were <5) were employed. The odds ratio (OR) with the 95% CI was estimated as the measure of association. Multiple testing in each family of tests (12 demographic and clinical characteristics, 14 volume outcomes) was corrected using the false discovery rate linear step-up procedure (32). The Benjamini–Hochberg critical values were calculated as *(i/m)Q*, where *i* is the rank in an ascending list of *p*-values, *m* is the total number of tests, and *Q* is a false discovery rate of 0.05. Similarly, after significant global tests, *post-hoc* tests were conducted using the Benjamini–Hochberg method. All the raw *p*-values were two-tailed. Statistical evaluation was performed using the statistical package SAS version 9.4 (SAS Institute, Cary, NC) and the R Statistical Software (version 4.0.2).

For the volume of the segmented structures, the mean (range) inter-observer variation was 3.3% (2.2–5.4%), and the mean (range) intra-observer variation was 4.4% (1.8–6.4%). For the size of the thalamus, the mean (range) inter-observer variation was even smaller, at 1.8% (0.4–3.3%) and the mean (range) of intra-observer variation was 3.0% (1.5–4.4%).

### Ethics

The study was approved by the Research Ethics Committee of the University of Tartu. Written informed consent was provided by all individual participants older than 7 years who were able to read, as well as by their parents.

## Results

### Population

**Figure 1:**
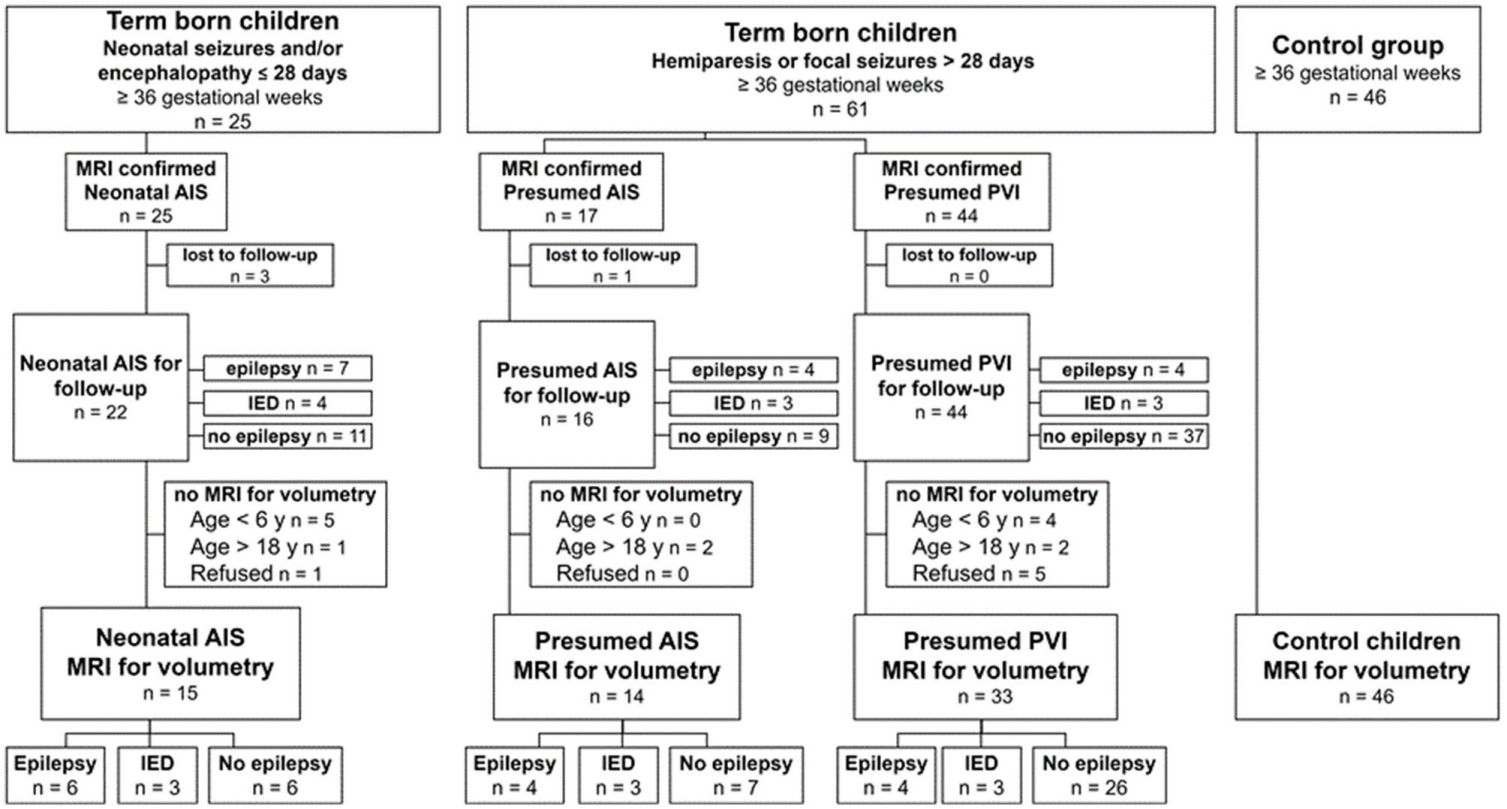
Patient selection.

The initial cohort of children with ischemic perinatal stroke included 86 children. Twenty-four patients were excluded because of being lost to follow-up or the absence of MRI, according to the terms of the study. The flow chart of the study population is presented in Figure 1. The final study group consisted of 46 controls and 62 patients with perinatal stroke: 15 children had neonatal AIS, 14 children had presumed perinatal AIS, and 33 children had presumed PVI.

All demographic and clinical data for the study groups are provided in Table 1. There were no differences in sex, gestational age, median age at follow-up MRI, or median age at the last clinical follow-up between the patients of the AIS, PVI, and control groups (Table 1). Similarly, the PSOM score did not differ between the AIS and PVI groups. AIS was left-sided in 72.4% and PVI in 48.5% of the children (*p* = 0.055). Patients with AIS had lower Apgar scores at the first and fifth min (*p* < 0.001) compared to patients in the PVI group. Neonatal seizures appeared in 10 of 15 (66.7%) children with neonatal AIS.

**Table 1:** Demographics and clinical characteristics.

	**AIS (*n* = 29)**	**PVI (*n* = 33)**	**Control (*n* = 46)**	**Overall *p***
Male sex, *n* (%)	15 (52)	14 (42)	27 (59)	0.36
Gestational weeks at birth, median (IQR)	40 (38–40)	40 (39,40)	NA	0.44
Apgar score at 1 min, median (IQR)	8 (5–8)	8 (8–9)	NA	<0.0001^*^
Apgar score at 5 min, median (IQR)	8 (7–9)	9 (9)	NA	<0.001^*^
Emergency cesarean section *n* (%)	12/28^**^ (43)	5 (15)	NA	0.016^*^
Neonatal seizures in NAIS, *n* (%)	10/15 (67)	NA	NA	NA
Side of stroke left, *n* (%)	21 (72)	16 (49)	NA	0.055
Age at follow-up MRI for volumetry, years, median (IQR)	10.7(8.5–14.1)	10.1 (7.7–12.8)	10.7 (9.6–13.1)	0.49
Age at last follow-up, years, median (IQR)	12.8 (10.8–17.3)	12.5 (9.3–14.8)	NA	0.32
Epilepsy, *n* (%)	10 (34.5)	4 (12.1)		0.036
Focal onset seizures, *n* (%)	9/10 (90)	4/4 (100)		>0.99
Epileptic spasms, *n* (%)	2/10 (20)	0/4 (0)		>0.99
Electrical status epilepticus in sleep, *n* (%)	1/10 (10)	1/4 (25)		0.51
Status epilepticus, *n* (%)	1/10 (10)	2/4 (50)		0.18
Polytherapy, *n* (%)	5/10 (50)	3/4 (75)		0.58
IED on postneonatal EEG without epilepsy, *n* (%)	6 (20.7)	3 (9.1)		0.28
Epilepsy or IED in postneonatal EEG, *n* (%)	16 (55.2)	7 (21.2)		0.0057^*^
PSOM score, median (IQR)	2.5 (1.5–4.0)	2.0 (1.5–2.5)		0.12

IED, interictal epileptiform discharges; PSOM, Pediatric Stroke Outcome Measure; *n*, number of observations; IQR, interquartile range; *N*/A, not applicable.

** For a single adopted child, the delivery mode was not known. Only the *p*-values that are below the significance threshold of the adjusted false discovery rate (0.0167) are significant and marked with an asterisk (^*^).

### Epilepsy

Epilepsy developed in 10 of 29 (34.5%) AIS patients and in 4 of 33 (12.1%) PVI patients (*p* = 0.036, OR = 3.8 95% CI: 1.04–14). Without epileptic seizures, IED occurred additionally in 20.7% (*n* = 6) of the AIS patients and in 9.1% (*n* = 3) of the PVI patients (*p* = 0.28) (Table 1; Figure 1). Overall, epilepsy or IED occurred more often in patients with AIS (*n* = 16, 55.2%) than in patients with PVI (*n* = 7, 21.2%) (*p* = 0.0057, OR = 3.8 95% CI: 1.04–14). The median time for the first epileptic seizure was not different between AIS patients and PVI patients, 4.8 and 3.3 years, respectively, (*p* = 0.62), with a median follow-up time of 12.8 and 12.5 years, respectively (*p* = 0.32).

#### Size of the thalamus and basal ganglia in the AIS, PVI, and control groups

There were no differences in the size of the ipsilesional and contralesional subcortical structures between the groups of neonatal AIS and presumed perinatal AIS; the data are provided in Supplementary Table S1. Therefore, in further analysis, the groups of neonatal AIS and presumed perinatal AIS were considered together under the AIS group.

Both children with AIS and PVI had a smaller ipsilesional thalamus, putamen, globus pallidus, and nucleus accumbens compared to controls. Additionally, children with AIS had a smaller ipsilesional hippocampus and a larger contralesional putamen compared to controls. Children with PVI also had a smaller ipsilesional caudate nucleus compared to controls (Table 2).

**Table 2:** Volume of the normalized subcortical brain structures in the AIS, PVI, and control groups.

	**AIS (*n* = 29)**	**PVI (*n* = 33)**	**Control (*n* = 46)**	**Overall *p***
Scaling factor	1.55 (1.46–1.64)	1.49 (1.43–1.55)	1.38 (1.35–1.42)	0.0007^bc^
**Ipsilesional**				
Thalamus	8,220 (7,102–9,338)	9,046 (8,284–9,808)	11,456 (11,232–11,680)	<0.0001^bc^
Caudate nucleus	5,595 (4,264, 6,129)	5,052 (4,550, 5,598)	5,818 (5,457, 6,115)	0.0001^c^
Putamen	6,323 (3,480, 7,786)	6,908 (5,931, 7,612)	7,289 (6,976, 7,791)	0.010^bc^
Globus pallidus	2,137 (1,489, 2,373)	2,219 (2,005, 2,381)	2,451 (2,321, 2,535)	<0.0001^bc^
Hippocampus	4,600 (4,052, 5,124)	5,026 (4,436, 5,394)	5,230 (4,993, 5,425)	0.0017^b^
Amygdala	1,390 (1,253–1,527)	1,537 (1,411–1,663)	1,592 (1,493–1,690)	0.051
Nucleus accumbens	575 (482–667)	624 (545–702)	747 (703–792)	0.0009^bc^
**Contralesional**				
Thalamus	10,602 (10,148–11,057)	10,760 (10,349–11,172)	11,060 (10,847–11,274)	0.14
Caudate nucleus	5,924 (5,446, 6,601)	5,795 (5,389, 6,387)	6,029 (5,472, 6,228)	0.64
Putamen	7,891 (7,559–8,223)	7,150 (6,820–7,480)	7,277 (7,089–7,465)	0.0008^ab^
Globus pallidus	2,486 (2,374–2,597)	2,369 (2,250–2,489)	2,496 (2,442–2,549)	0.094
Hippocampus	5,388 (5,133–5,644)	5,171 (4,928–5,415)	5,296 (5,148–5,444)	0.37
Amygdala	1,465 (1,320–1,609)	1,672 (1,540–1,804)	1,538 (1,457–1,619)	0.049
Nucleus accumbens	658 (586–731)	600 (552–649)	608 (573–643)	0.24

The data are presented in mm^3^: mean with the 95% confidence interval [e.g., X (Y–Z)] or median with the 25th and 75th percentiles [e.g., X (Y, Z)] as statistically appropriate; only the overall *p*-values that are below the significance threshold of the adjusted false discovery rate (0.027) are significant. Pairwise comparisons were conducted using the Benjamini–Hochberg method, and only the *p*-values that are below the significance threshold of the adjusted false discovery rate are therefore significant and marked with a superscript: ^a^AIS vs. PVI, ^b^AIS vs. control, and ^c^PVI vs. control.

There were no differences in the size of the subcortical structures between children with AIS and PVI, except for children with AIS, who had a larger size of the contralesional putamen compared to children with PVI (Table 2).

#### Size of the thalamus and basal ganglia in the groups of AIS and PVI with epilepsy

**Figure 2:**
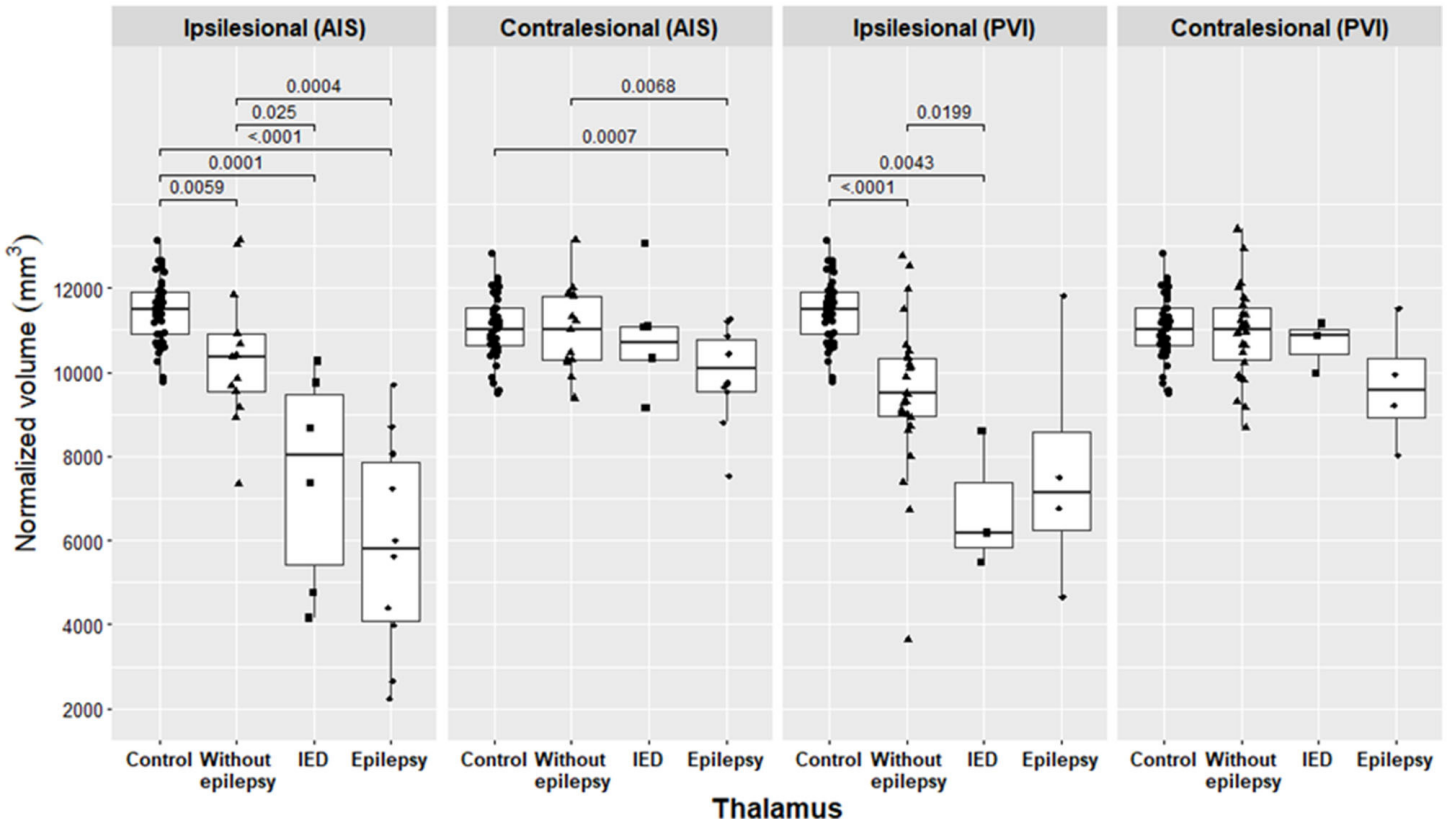
Volume of the normalized thalamus in the AIS and PVI subgroups and in the control group. Pairwise comparisons were conducted using the Benjamini–Hochberg method, and only the *p*-values that are below the significance threshold of the adjusted false discovery rate are significant and presented in the figure.

The ipsilesional thalamus was smaller in children of the AIS group with epilepsy and in children of the pooled group of epilepsy + IED either with AIS or PVI compared to children without epilepsy (Figure 2; Tables 3, 4). The contralesional thalamus was only smaller in children of the AIS group with epilepsy compared to children without epilepsy, but not in children of the PVI group. The caudate nucleus and nucleus accumbens were smaller in children of the AIS group with epilepsy compared to children without epilepsy (Supplementary Figures S1, S6).

**Table 3:** Volume of the normalized subcortical brain structures in the AIS and control groups.

	**Control**	**AIS**				**AIS**	
	**(*****n*** = **46)**	**Without epilepsy (*****n*** = **13)**	**IED (*****n*** = **6)**	**Epilepsy (*****n*** = **10)**	**Overall** *p*^1^	**Epilepsy** + **IED (*****n*** = **16)**	**Overall** *p*^2^
Scaling factor	1.41 (1.32, 1.44)	1.36 (1.29, 1.59)	1.59 (1.46, 1.68)	1.57 (1.43, 1.70)	0.0009^bc^	1.59 (1.46, 1.69)	0.0046^xy^
**Ipsilesional**							
Thalamus	11,508 (10,905, 11,906)	10,362 (9,532, 10,897)	8,016 (4,766, 9,746)	5,805 (3,996, 8,065)	<0.0001^abcde^	6,614 (4,278, 8,693)	<0.0001^xyz^
Caudate nucleus	5,818 (5,457, 6,115)	5,755 (5,079, 6,138)	6,074 (5,637, 6,340)	3,898 (2,179, 5,214)	0.0021^cef^	5,181 (2,308, 5,943)	0.045
Putamen	7,289 (6,976, 7,791)	7,161 (6,005, 7,953)	6,407 (3,480, 7,871)	3,892 (1,770, 6,323)	0.0086^c^	5,616 (1,811, 7,576)	0.0071^y^
Globus pallidus	2,451 (2,321, 2,535)	2,373 (2,093, 2,517)	2,105 (1,489, 2,353)	1,741 (343, 2,359)	0.0001^bc^	2,072 (632, 2,355)	<0.0001^yz^
Hippocampus	5,230 (4,993, 5,425)	4,994 (4,401, 5,306)	4,326 (3,217, 5,178)	4,131 (3,439, 4,817)	0.0007^c^	4,131 (3,348, 4,851)	0.0003^yz^
Amygdala	1,621 (1,350, 1,817)	1,571 (1,354, 1,699)	1,079 (835, 1,480)	1,316 (1,190, 1,645)	0.0038^bd^	1,271 (935, 1,518)	0.0046^yz^
Nucleus accumbens	765 (680, 859)	685 (588, 810)	468 (273, 597)	450 (360, 526)	0.0001^bcde^	456 (357, 545)	<0.0001^yz^
**Contralesional**							
Thalamus	11,024 (10,610, 11,527)	11,000 (10,290, 11,823)	10,711 (10,301, 11,084)	10,083 (9,526, 10,871)	0.0075^ce^	10,380 (9,590, 11,082)	0.028^y^
Caudate nucleus	6,029 (5,472, 6,228)	5,861 (5,586, 6,416)	6,725 (6,239, 6,868)	5,685 (5,214, 6,180)	0.016^bdf^	6,150 (5,380, 6,648)	0.65
Putamen	7,353 (6,730, 7,779)	7,692 (7,020, 8,460)	8,572 (8,312, 9,196)	7,793 (7,195, 8,139)	0.0003^bdf^	8,123 (7,416, 8,572)	0.0013^y^
Globus pallidus	2,516 (2,375, 2,626)	2,492 (2,385, 2,693)	2,481 (2,336, 2,546)	2,564 (2,053, 2,698)	0.95	2,542 (2,248, 2,642)	0.86
Hippocampus	5,251 (4,989, 5,610)	5,358 (4,772, 5,844)	5,550 (4,867, 6,081)	5,369 (5,234, 5,806)	0.59	5,369 (5,087, 6,004)	0.64
Amygdala	1,515 (1,382, 1,697)	1,603 (1,404, 1,936)	1,222 (1,129, 1,397)	1,445 (1,275, 1,627)	0.31	1,350 (1,164, 1,567)	0.14
Nucleus accumbens	592 (534, 691)	686 (666, 786)	580 (525, 700)	597 (475, 743)	0.16	580 (487, 721)	0.08

The data are presented as median (IQR) in mm^3^.

Overall *p*^1^–comparison between children with epilepsy, without epilepsy, with IED, and controls. Only the overall *p*-values that are below the significance threshold of the adjusted false discovery rate (0.037) are significant. Pairwise comparisons were conducted using the Benjamini–Hochberg method, and only the *p*-values that are below the significance threshold of the adjusted false discovery rate are significant and marked with a superscript: ^a^control vs. without epilepsy; ^b^control vs. IED; ^c^control vs. epilepsy; ^d^without epilepsy vs. IED; ^e^without epilepsy vs. epilepsy; and ^f^epilepsy vs. IED.

Overall *p*^2^–comparison between children without epilepsy, with both epilepsy + IED, and controls. Only the *p*-values that are below the significance threshold of the adjusted false discovery rate (0.030) are significant. Pairwise comparisons were conducted using the Benjamini–Hochberg method, and only the *p*-values that are below the significance threshold of the adjusted false discovery rate are significant and marked with a superscript: ^x^control vs. without epilepsy; ^y^control vs. epilepsy + IED; and ^z^without epilepsy vs. epilepsy + IED.

**Table 4:** Volume of the normalized subcortical brain structures in the PVI and control groups.

	**Control**	**PVI**				**PVI**	
	**(*****n*** = **46)**	**Without epilepsy (*****n*** = **26)**	**IED (*****n*** = **3)**	**Epilepsy (*****n*** = **4)**	**Overall** *p*^1^	**Epilepsy** + **IED (*****n*** = **7)**	**Overall** *p*^2^
Scaling factor	1.41 (1.32, 1.44)	1.47 (1.38, 1.56)	1.50 (1.46, 1.87)	1.58 (1.46, 1.87)	0.092	1.50 (1.42, 1.67)	0.017^x^
**Ipsilesional**							
Thalamus	11,508 (10,905, 11,906)	9,494 (8,937, 10,350)	6,177 (5,474, 8,589)	7,140 (5,716, 9,669)	<0.0001^abd^	6,771 (5,474, 8,589)	<0.0001^xyz^
Caudate nucleus	5,818 (5,457, 6,115)	5,128 (4,725, 5,664)	3,788 (2,460, 5,792)	3,905 (2,109, 4,713)	<0.0001^ac^	3,793 (2,460, 5,410)	<0.0001^xyz^
Putamen	7,289 (6,976, 7,791)	7,028 (6,188, 7,758)	5,931 (3,132, 7,490)	5,304 (3,240, 6,884)	0.032	5,931 (3,132, 7,271)	0.013^y^
Globus pallidus	2,451 (2,321, 2,535)	2,273 (2,064, 2,418)	2,118 (907, 2,374)	1,931 (1,217, 2,274)	0.0005^ac^	2,118 (907, 2,367)	0.0001^xy^
Hippocampus	5,230 (4,993, 5,425)	5,046 (4,436, 5,416)	4,459 (4,056, 4,711)	4,230 (2,798, 5,398)	0.076	4,459 (3,435, 5,026)	0.042
Amygdala	1,621 (1,350, 1,817)	1,538 (1,348, 1,889)	1,080 (997, 1,344)	1,640 (1,231, 1,964)	0.17	1,344 (997, 1,759)	0.39
Nucleus accumbens	765 (680, 859)	693 (572, 844)	480 (295, 620)	452 (315, 639)	0.0024^bc^	476 (295, 620)	0.0007^yz^
**Contralesional**							
Thalamus	11,024 (10,610, 11,527)	11,004 (10,227, 11,587)	10,875 (9,969, 11,153)	9,573 (8,612, 10,727)	0.25	9,969 (9,206, 11,153)	0.17
Caudate nucleus	6,029 (5,472, 6,228)	5,931 (5,389, 6,441)	6,387 (5,627, 6,841)	5,284 (2,871, 5,605)	0.13	5,627 (5,154, 6,387)	0.73
Putamen	7,353 (6,730, 7,779)	7,181 (6,423, 7,639)	8,280 (7,454, 8,344)	6,637 (6,082, 7,383)	0.08	7,454 (6,392, 8,280)	0.49
Globus pallidus	2,516 (2,375, 2,626)	2,411 (2,218, 2,525)	2,468 (2,337, 2,612)	2,170 (1,572, 2,482)	0.10	2,356 (1,984, 2,608)	0.075
Hippocampus	5,251 (4,989, 5,610)	5,210 (4,907, 5,678)	4,907 (4,879, 5,258)	5,172 (4,368, 5,744)	0.63	4,907 (4,716, 5,628)	0.53
Amygdala	1,515 (1,382, 1,697)	1,620 (1,460, 1,925)	1,426 (1,032, 1,762)	2,088 (1,828, 2,176)	0.019	1,762 (1,426, 2,154)	0.15
Nucleus accumbens	592 (534, 691)	664 (523, 707)	604 (582, 724)	469 (392, 537)	0.14	545 (408, 604)	0.33

The data are presented as median (IQR) in mm^3^.

Overall *p*^1^–comparison between children with epilepsy, without epilepsy, with IED, and controls. Only the overall *p*-values that are below the significance threshold of the adjusted false discovery rate (0.013) are significant. Pairwise comparisons were conducted using the Benjamini–Hochberg method, and only the *p*-values that are below the significance threshold of the adjusted false discovery rate are significant and marked with a superscript: ^a^control vs. without epilepsy; ^b^control vs. IED; ^c^control vs. epilepsy; ^d^without epilepsy vs. IED; ^e^without epilepsy vs. epilepsy; and ^f^epilepsy vs. IED.

Overall *p*^2^–comparison between children without epilepsy, with both epilepsy+IED, and controls. Only the *p*-values that are below the significance threshold of the adjusted false discovery rate (0.020) are significant. Pairwise comparisons were conducted using the Benjamini–Hochberg method, and only the *p*-values that are below the significance threshold of the adjusted false discovery rate are significant and marked with a superscript: ^x^control vs. without epilepsy; ^y^control vs. epilepsy + IED; and ^z^without epilepsy vs. epilepsy + IED.

**Figure 3:**
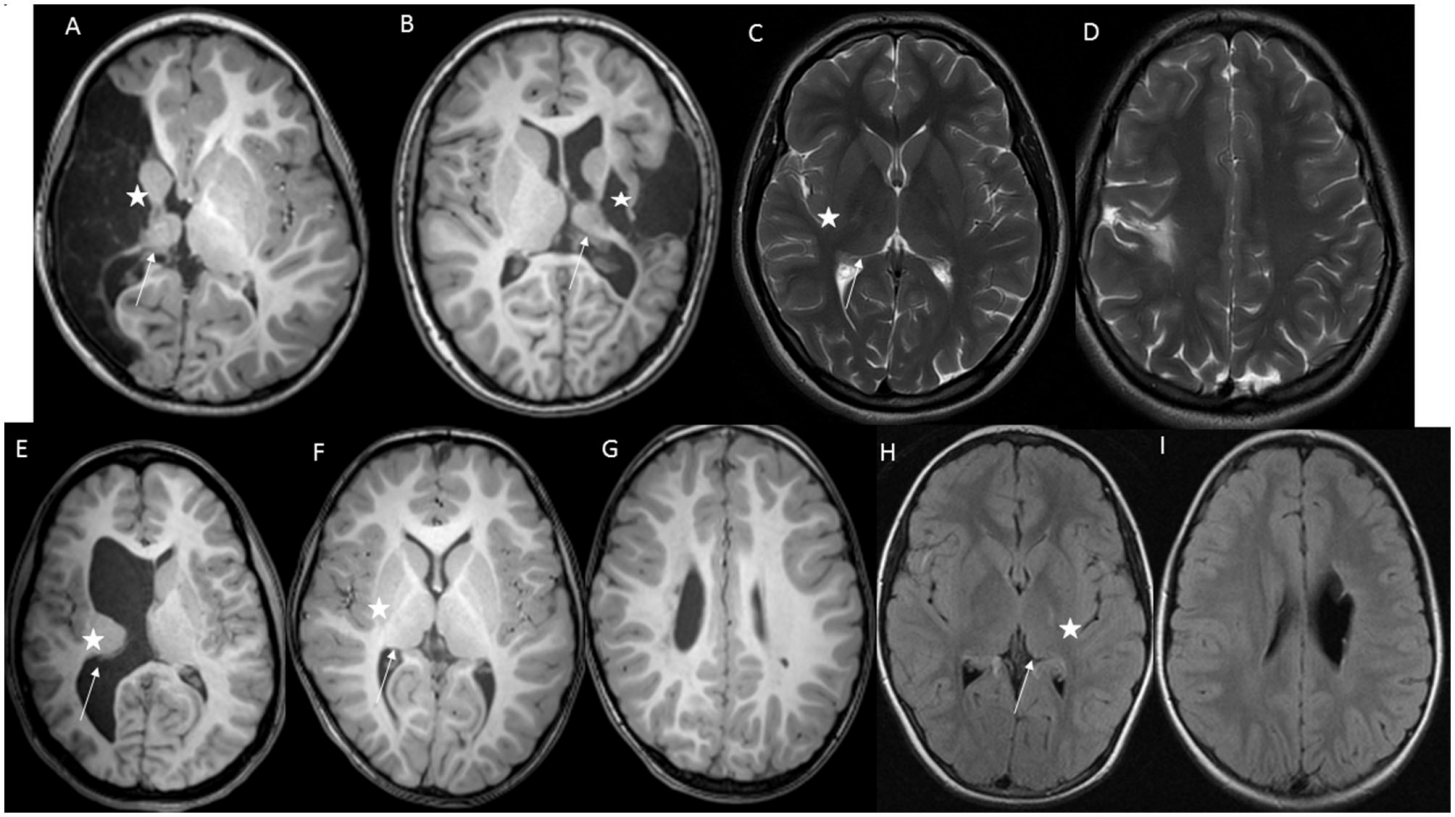
Individual MRI findings in children with perinatal stroke. Basal ganglia are marked with an asterisk, and the thalamus is marked with an arrow. **(A)** A girl at age 5 with neonatal AIS, right-side proximal middle cerebral artery stroke, and epilepsy. Axial T1-weighted MRI image shows the ipsilesional small size of the basal ganglia and thalamus. **(B)** A boy at age 14 with neonatal AIS and left-side proximal middle cerebral artery stroke, and IED. Axial T1-weighted MRI image shows the ipsilesional small size of the basal ganglia and thalamus. **(C, D)** A girl at age 16 with neonatal AIS and left-side anterior trunk of middle cerebral artery stroke without epilepsy. Axial PROPELLER T2-weighted MRI image shows the ipsilesional normal size of the basal ganglia and thalamus. **(E)** A girl at age 17 with large right-side presumed PVI and epilepsy. Axial T1-weighted MRI image shows right-side periventricular damage and ipsilesional small size of the basal ganglia and thalamus. **(F, G)** A girl at age 7 with right-side presumed PVI and IED. Axial T1-weighted MRI image shows right-side periventricular damage and ipsilesional small size of the thalamus and normal basal ganglia. **(H, I)** A girl at age 11 with left-side presumed PVI and without epilepsy. Axial FLAIR MRI image shows left-side periventricular damage and ipsilesional minimally smaller size of the thalamus and normal basal ganglia.

In the pooled group of epilepsy + IED, both children with AIS or PVI had, in addition to the smaller thalamus, a smaller nucleus accumbens compared to children without epilepsy. In the pooled group of epilepsy + IED, patients with AIS also had a smaller globus pallidus, hippocampus, and amygdala, and patients with PVI had a smaller caudate nucleus compared to children without epilepsy. Individual MRI findings in children of the AIS group with and without epilepsy (A, C, and D) and of the PVI group with and without epilepsy (E, H, and I) are presented in Figure 3.

#### Size of the thalamus and basal ganglia in the groups of AIS and PVI with IED

The ipsilesional thalamus was smaller in children with IED and in patients with AIS or PVI compared to children without epilepsy (Figure 2; Tables 3, 4). In children in the AIS group with IED, the ipsilesional amygdala and nucleus accumbens were smaller, and the contralesional caudate nucleus and putamen were larger compared to children without epilepsy (Supplementary Figures S1, S2, S5, S6).

In children with PVI and IED, the subcortical structures did not differ from those of children with epilepsy. Children in the AIS group with IED had a larger ipsilesional and contralesional caudate nucleus and contralesional putamen compared to children with epilepsy (Supplementary Figures S1, S2). Individual MRI findings in children with AIS and IED (B) and with PVI and IED (F, G) are presented in Figure 3.

#### Size of the thalamus and basal ganglia in the groups of AIS and PVI with and without epilepsy compared to controls

In the AIS group, all ipsilesional subcortical structures, except for the amygdala in children with epilepsy and the caudate nucleus in the pooled group of epilepsy + IED, were smaller compared to controls (Figure 2; Tables 3, 4; Supplementary Figures S1–S6). In the AIS group, the contralesional thalamus was smaller in children with epilepsy and in children of the pooled group of epilepsy + IED, and the putamen was larger in the pooled group compared to controls. Without epilepsy in the AIS group, only the ipsilesional thalamus was smaller compared to controls.

In the PVI group, the ipsilesional caudate nucleus, globus pallidus, and nucleus accumbens were smaller in children with epilepsy and in children of the pooled group compared to controls. In the pooled group, the ipsilesional thalamus and putamen were smaller compared to controls. In children without epilepsy, the ipsilesional thalamus, caudate nucleus, and globus pallidus were smaller compared to controls.

## Discussion

This study provides the first comprehensive assessment of the size of the thalamus and basal ganglia in children with ischemic perinatal stroke and epilepsy. We found significant differences in the volume of the thalamus and basal ganglia between those who developed epilepsy and those without epilepsy. At the same time, different patterns of change in the basal ganglia and hippocampus were revealed in the analysis of the AIS and PVI groups, as well as their different subgroups.

Our study showed that epilepsy or IED occurred more often among children with AIS compared to children with PVI. The ipsilesional thalamus was smaller in children of the AIS group with epilepsy, in children of the AIS and PVI groups with IED, and in children of the pooled group of epilepsy + IED compared to children without epilepsy. The contralesional thalamus was only smaller in children in the AIS group with epilepsy compared to children without epilepsy. In addition to the thalamus, the caudate nucleus and nucleus accumbens were smaller in children in the AIS group with epilepsy compared to children without epilepsy.

To date, there is no clear understanding regarding which children with perinatal AIS or PVI are at a higher risk of developing epilepsy. In several studies, family history of seizures, cerebral palsy, newborn seizures, initial presentation with cognitive impairment, or evidence of infarction on prenatal ultrasound have been considered risk factors for epilepsy (12, 33, 34). Some studies claim that the risk of seizures is higher among those with larger strokes or multiple strokes (8, 9, 18, 35). In some vascular subtypes of perinatal stroke, epilepsy may develop more frequently, and children with AIS are affected the most (3, 8).

In previous studies of children with ischemic perinatal stroke, changes in the volume of the subcortical structures have been described mainly in association with motor function (16, 17). Some studies have also ascribed changes in the volume of the basal ganglia, thalamus, and hippocampus to the relationship with poststroke epilepsy. In children with AIS, a significant reduction in hippocampal volume was found in patients with seizures compared to controls, which was not observed in patients without seizures (20). Research into infantile spasms after perinatal AIS indicates that injury to the deep gray structures, i.e., the basal ganglia and the thalamus, may be important for the development of infantile spasms (19).

According to our study, the smaller volume of the subcortical structures may predict which children will be at the greatest risk of epilepsy after ischemic perinatal stroke. More extensive changes in the volume of the subcortical structures in patients with epilepsy were observed in the AIS group. Besides the smaller ipsilesional and contralesional thalamus, the ipsilesional caudate nucleus and nucleus accumbens also were smaller compared to children without epilepsy. The size of the subcortical structures of children with PVI did not differ between the subgroups of epilepsy, IED, or without epilepsy, except for the smaller ipsilesional thalamus in the IED group compared to children without epilepsy. As the groups of children with epilepsy and with IED alone were small, we pooled these children into one group. In this case, we found that, compared to children without epilepsy, the thalamus and the other subcortical structures were also smaller in the PVI group. The fact that changes in the subcortical structures were less pronounced in children with PVI may explain why these children develop epilepsy less frequently.

Our results, which also showed changes in the contralesional thalamus in children with epilepsy, are concordant with growing evidence about unilateral or bilateral thalamic involvement in networks in different types of epilepsy. In adults with mesial temporal lobe epilepsy, analysis of thalamic volume has revealed bilateral thalamic abnormalities (36, 37). A study of patients with different medically intractable focal epilepsies, including childhood-onset epilepsies, found changes in tissue properties in the otherwise normal appearing thalamus, pallidum, caudate nucleus, and nucleus accumbens, with ipsilateral predominance and thalamic preference (38). Regarding childhood epilepsies, changes in bilateral thalamic and other deep brain structures are described in generalized epilepsy syndromes, localized epilepsies, and self-limited childhood epilepsies (39, 40).

Apart from the thalamus, we also found other structures with contralesional changes, indicating the bilateral reorganization of the brain after perinatal stroke. A significantly larger size of the contralesional putamen and caudate nucleus was noticed in children with IED, compared not only to controls and children without epilepsy but even to children with epilepsy among those with AIS. The clinical importance of these contralesional changes is not clear, but a similar trend was also evident among children with PVI.

The similarities between children with epilepsy and those with IEDs were the most intriguing. In the AIS group, only the contralesional caudate nucleus and putamen and the ipsilesional caudate nucleus were smaller in children with epilepsy compared to children with IED. In the PVI group, the size of the thalamus and basal ganglia, both ipsilesionaland contralesionally, did not differ between children with IED and those with epilepsy.

Previous functional studies of seizures have shown the involvement of the caudate nucleus and putamen in generating ictal activity during epileptic seizures (41, 42). We support this idea and speculate that extensive damage to the thalamus, accompanied by damage to the caudate nucleus and putamen, may play a role in the development of epilepsy after ischemic perinatal stroke, especially without the concurrent contralesional enlargement of the caudate nucleus and putamen as seen in children with IED. However, epilepsy in children with perinatal stroke may develop until young adulthood (11), and similarities in the size of the basal ganglia and thalamus between children with epilepsy and those with IED suggest that longer follow-up studies are needed to understand if children with IED develop epilepsy later. Therefore, regular EEG evaluations after perinatal stroke are needed, at least in children with AIS, to identify possible epileptiform discharges.

It is also important to stress the differences between the control group and patients with AIS or PVI with epilepsy. In children with epilepsy, changes in the volume of the subcortical structures involved more structures compared to controls than in children without epilepsy. Moreover, in the AIS group, only the thalamus was smaller in children without epilepsy compared to controls, and volume changes were not as extensive as in the epilepsy group.

Our findings indicate that it is important to perform a follow-up MRI after the acute period of stroke and to evaluate the size of the thalamus and the basal ganglia, which provides information not only about motor outcome and hand function but also about the risk of epilepsy. Patients with severe damage to the basal ganglia and the thalamus are at higher risk for the development of poststroke epilepsy and should be closely monitored throughout childhood in order to start with timely antiseizure medication and rehabilitation.

A strength of our study is the long follow-up time compared to other studies (5, 6, 35). We did not exclude children without epilepsy diagnosis who still had IED, but analyzed them under a separate group, as we did not know the importance of the finding in terms of outcome and possibility for the development of epilepsy. Another strength of our study is the inclusion of children with both perinatal AIS and PVI, which were the most frequent vascular subtypes of perinatal stroke, and the inclusion of cases with various degrees of damage.

The volume of the subcortical structures was analyzed using volumetric analysis by segmentation with the FSL FIRST tool. Manual segmentation was needed to correct the faults induced by stroke-related large morphological changes in automatic segmentation. This could be an advantage and a disadvantage at the same time due to human error. To reduce the hazard of bias, inter- and intra-rater reliabilities were estimated with high agreement between measurements, especially in the case of measuring the size of the thalamus.

This study has some limitations. The study group was relatively small to find all possibly relevant differences between the subgroups, in particular, considering children with IEDs alone. In addition, we could not use a standardized age at which EEG and follow-up MRI were performed. However, the age at the last clinical follow-up did not differ between children with epilepsy and those with IED. Furthermore, as there was a gap between the first epileptic seizure and the follow-up MRI, we cannot exclude the impact of epilepsy itself on the volume of the subcortical structures. Even though we had a longer follow-up time compared with many other studies, it still covered the period until early teenage age. Thus, we are unaware pf how many more children will develop epilepsy in future. It should be noted that we were unable to enroll all patients in the Estonian Pediatric Stroke Database due to either refusal or non-availability. Further prospective multicenter and longitudinal studies are needed to verify all relevant changes in the subcortical structures, which could determine the development of poststroke epilepsy.

## Conclusion

Epilepsy or IED developed more often in children with AIS compared to children with PVI. Patients with severe damage not only to the thalamus but also to the basal ganglia are at a higher risk for the development of poststroke epilepsy. Our data highlight the importance of follow-up radiological evaluation of the thalamus and the basal ganglia in predicting the risk of epilepsy and timely interventions. The findings of this study will contribute to our understanding of the development of poststroke epilepsy in perinatal stroke and of the overall epilepsy network.

## Data availability statement

The raw data supporting the conclusions of this article will be made available by the authors, without undue reservation.

## Ethics statement

The studies involving humans were approved by Research Ethics Committee of the University of Tartu, Estonia. The studies were conducted in accordance with the local legislation and institutional requirements. Written informed consent for participation in this study was provided by the participants' legal guardians/next of kin.

## Author contributions

UV, PI, NiI, and MM: conceptualization and methodology. UV, NiI, NoI, and PI: data curation. UV, NiI, NoI, RL, DL, and PI: investigations. NiI and PK: software. PI: resources. PK: formal analysis and visualization. PI and MM: supervision. All authors contributed to the writing of the manuscript and approved its final version.
