# Supplementary material for: The thalamus and basal ganglia are smaller in children with epilepsy after perinatal stroke
Source: Front Neurol. 2023 Sep 28;14:1252472. doi: 10.3389/fneur.2023.1252472 (PMC10568465; doi:10.3389/fneur.2023.1252472)
Supplement: Supplementary file 2 [file Image_2.pdf]

Supplementary Figure S2. Volume of the normalized putamen in the AIS and PVI subgroups and in the control group.

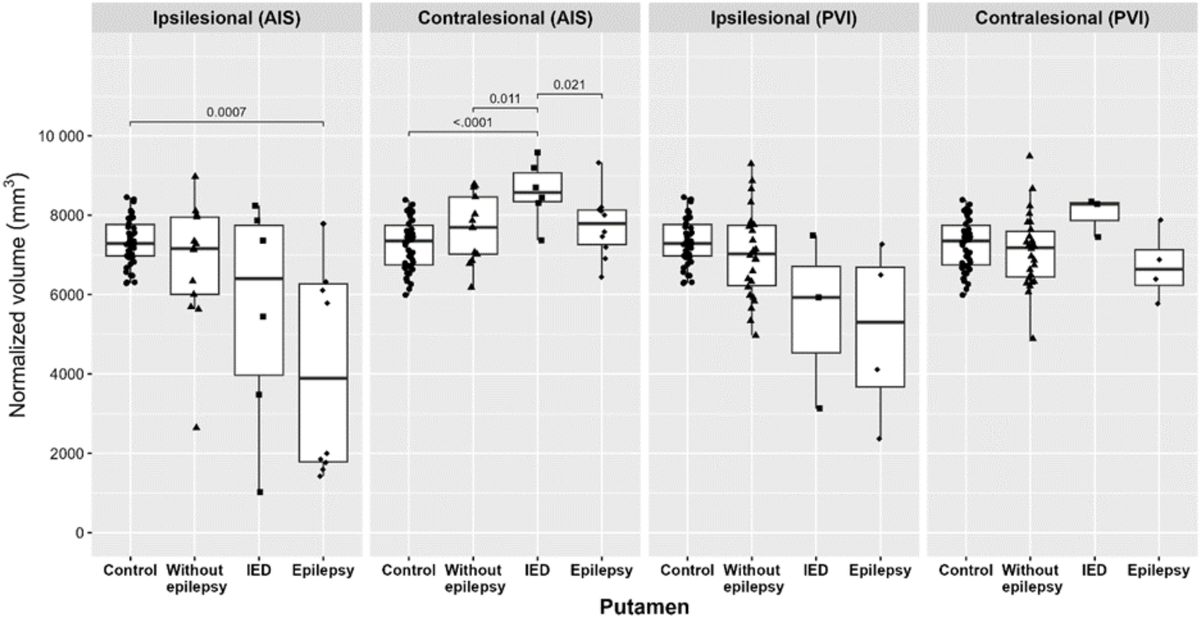

Pairwise comparisons were conducted using the Benjamini-Hochberg method and only the p values that are below the significance threshold of the adjusted false discovery rate are significant and presented in the figure.
