# Supplementary material for: The thalamus and basal ganglia are smaller in children with epilepsy after perinatal stroke
Source: Front Neurol. 2023 Sep 28;14:1252472. doi: 10.3389/fneur.2023.1252472 (PMC10568465; doi:10.3389/fneur.2023.1252472)
Supplement: Supplementary file 7 [file Table_1.pdf]

Table S1. Volume of the normalized subcortical brain structures in the AIS and PVI groups.

	NAIS (n=15)	PAIS (n=14)	P value
**Ipsilesional**			
**Thalamus**	8019 (6143-9895)	8435 (7003-9868)	0.71
**Nucleus caudatus**	5388 [2916, 6138]	5654 [4753, 6129]	0.65
**Putamen**	6109 [2001, 7786]	6736 [5449, 7786]	0.53
**Globus pallidus**	2032 [1370, 2373]	2266 [2093, 2453]	0.28
**Hippocampus**	4340 (3764-4916)	4621 (4177-5066)	0.42
**Amygdala**	1332 (1114-1551)	1451 (1266-1637)	0.38
**Nucleus accumbens**	456 [360, 685]	607 [480, 769]	0.33
**Contralesional**			
**Thalamus**	10637 (10046-11227)	10565 (9781-11350)	0.88
**Nucleus caudatus**	6109 (5665-6553)	6021 (5599-6442)	0.76
**Putamen**	7956 (7567-8345)	7822 (7217-8427)	0.69
**Globus pallidus**	2525 (2394-2656)	2443 (2242-2645)	0.47
**Hippocampus**	5286 (4923-5649)	5498 (5096-5901)	0.40
**Amygdala**	1410 (1214-1606)	1523 (1286-1760)	0.43
**Nucleus accumbens**	678 (554-802)	637 (547-727)	0.58

The data are presented in mm^3: mean with the 95% confidence interval [e.g. X (Y–Z)] or median with the 25th and 75th percentiles (e.g. X [Y, Z]) as statistically appropriate
